# Bupropion for the Treatment of Apathy in Alzheimer Disease: A Randomized Clinical Trial

**DOI:** 10.1001/jamanetworkopen.2020.6027

**Published:** 2020-05-28

**Authors:** Franziska Maier, Annika Spottke, Jan-Philipp Bach, Claudia Bartels, Katharina Buerger, Richard Dodel, Andreas Fellgiebel, Klaus Fliessbach, Lutz Frölich, Lucrezia Hausner, Martin Hellmich, Stefan Klöppel, Arne Klostermann, Johannes Kornhuber, Christoph Laske, Oliver Peters, Josef Priller, Tanja Richter-Schmidinger, Anja Schneider, Kija Shah-Hosseini, Stefan Teipel, Christine A. F. von Arnim, Jens Wiltfang, Frank Jessen

**Affiliations:** 1 Department of Psychiatry, University Hospital Cologne, Medical Faculty, Cologne, Germany; 2 German Center for Neurodegenerative Diseases, Bonn, Germany; 3 Department of Neurology, University of Bonn, Bonn, Germany; 4 Department of Geriatric Medicine, University Hospital Essen, Essen, Germany; 5 Department of Neurology, Philipps-University Marburg, Marburg, Germany; 6 Department of Psychiatry and Psychotherapy, University Medical Center Goettingen, Georg-August-University, Goettingen, Germany; 7 Institute for Stroke and Dementia Research, Klinikum der Universität München, Ludwig-Maximilians-Universität, Munich, Germany; 8 German Center for Neurodegenerative Diseases, Munich, Germany; 9 Center for Mental Health in Old Age, Landeskrankenhaus, Mainz, Germany; 10 Department of Geriatric Psychiatry, Zentralinstitut für Seelische Gesundheit Mannheim, University of Heidelberg, Mannheim, Germany; 11 Institute of Medical Statistics and Computational Biology, Faculty of Medicine, University of Cologne, Cologne, Germany; 12 University Hospital of Old Age Psychiatry, University of Bern, Bern, Switzerland; 13 German Center for Neurodegenerative Diseases, Berlin, Germany; 14 Department of Psychiatry, Charité Berlin, Berlin, Germany; 15 Department of Psychiatry and Psychotherapy, Friedrich Alexander University Erlangen-Nürnberg, Erlangen, Germany; 16 German Center for Neurodegenerative Diseases, Tübingen, Germany; 17 Section for Dementia Research, Hertie Institute for Clinical Brain Research, Department of Psychiatry and Psychotherapy, University of Tübingen, Tübingen, Germany; 18 Department of Neuropsychiatry, Berlin Institute of Health, Charité Berlin, Berlin, Germany; 19 Klinik für Neurodegenerative Erkrankungen und Gerontopsychiatrie, University of Bonn, Bonn, Germany; 20 German Center for Neurodegenerative Diseases, Rostock, Germany; 21 Department of Psychosomatic Medicine, University Hospital of Rostock, Rostock, Germany; 22 Department of Neurology, Ulm University Hospital, Ulm, Germany; 23 German Center for Neurodegenerative Diseases, Goettingen, Germany; 24 Excellence Cluster on Cellular Stress Responses in Aging-Associated Diseases, University of Cologne, Cologne, Germany

## Abstract

**Importance:**

Apathy is a frequent neuropsychiatric symptom in dementia of Alzheimer type and negatively affects the disease course and patients’ and caregivers’ quality of life. Effective treatment options are needed.

**Objective:**

To examine the efficacy and safety of the dopamine and noradrenaline reuptake inhibitor bupropion in the treatment of apathy in patients with dementia of Alzheimer type.

**Design, Setting, and Participants:**

This 12-week, multicenter, double-blind, placebo-controlled, randomized clinical trial was conducted in a psychiatric and neurological outpatient setting between July 2010 and July 2014 in Germany. Patients with mild-to-moderate dementia of Alzheimer type and clinically relevant apathy were included. Patients with additional clinically relevant depressed mood were excluded. Data analyses were performed between August 2018 and August 2019.

**Interventions:**

Patients received either bupropion or placebo (150 mg for 4 weeks plus 300 mg for 8 weeks). In case of intolerability of 300 mg, patients continued to receive 150 mg throughout the study.

**Main Outcomes and Measures:**

Change on the Apathy Evaluation Scale–Clinician Version (AES-C) (score range, 18-72 points) between baseline and week 12 was the primary outcome parameter. Secondary outcome parameters included measures of neuropsychiatric symptoms, cognition, activities of daily living, and quality of life. Outcome measures were assessed at baseline and at 4, 8, and 12 weeks.

**Results:**

A total of 108 patients (mean [SD] age, 74.8 [5.9] years; 67 men [62%]) were included in the intention-to-treat analysis, with 54 randomized to receive bupropion and 54 randomized to receive placebo. The baseline AES-C score was comparable between the bupropion group and the placebo group (mean [SD], 52.2 [8.7] vs 50.4 [8.2]). After controlling for the baseline AES-C score, site, and comedication with donepezil or galantamine, the mean change in the AES-C score between the bupropion and placebo groups was not statistically significant (mean change, 2.22; 95% CI, –0.47 to 4.91; *P* = .11). Results on secondary outcomes showed statistically significant differences between bupropion and placebo in terms of total neuropsychiatric symptoms (mean change, 5.52; 95% CI, 2.00 to 9.04; *P* = .003) and health-related quality of life (uncorrected for multiple comparisons; mean change, –1.66; 95% CI, –3.01 to –0.31; *P* = .02) with greater improvement in the placebo group. No statistically significant changes between groups were found for activities of daily living (mean change, –2.92; 95% CI, –5.89 to 0.06; *P* = .05) and cognition (mean change, –0.27; 95% CI, –3.26 to 2.73; *P* = .86). The numbers of adverse events (bupropion group, 39 patients [72.2%]; placebo group, 33 patients [61.1%]) and serious adverse events (bupropion group, 5 patients [9.3%]; placebo group, 2 patients [3.7%]) were comparable between groups.

**Conclusions and Relevance:**

Although it is safe, bupropion was not superior to placebo for the treatment of apathy in patients with dementia of Alzheimer type in the absence of clinically relevant depressed mood.

**Trial Registration:**

EU Clinical Trials Register Identifier: 2007-005352-17

## Key Points

**Question:**

Is bupropion an effective and safe treatment for apathy in nondepressed patients with dementia of Alzheimer type?

**Findings:**

In this 12-week, multicenter, double-blind, placebo-controlled, randomized clinical trial, 54 patients received bupropion and 54 received placebo. The mean change in the Apathy Evaluation Scale–Clinician Version score was not statistically significant between the treatment groups.

**Meaning:**

Bupropion did not improve apathy in patients with dementia of Alzheimer type without depressed mood.

## Teaser

This randomized clinical trial examines the efficacy and safety of the dopamine and noradrenaline reuptake inhibitor bupropion in the treatment of apathy in patients with dementia of Alzheimer type.

## Introduction

As the most frequent neuropsychiatric symptom in patients with dementia of Alzheimer type (DAT), apathy greatly affects the disease course, patients’ activities of daily living, and quality of life.^1,2,3^ Apathy increases caregiver burden^4^ and is associated with increased mortality.^5^ Apathy can occur during all stages of DAT and may even appear in the preclinical phase of Alzheimer disease (AD).^6^ Antidementia drugs, such as acetylcholinesterase inhibitors, are only of very limited efficacy in the treatment of apathy.^7^ A recent Cochrane review^8^ found only 4 randomized clinical trials with the primary goal of improving apathy. Thus, there is a need to improve treatment options.

The concept of apathy in DAT has been substantially developed over the last years.^9^ Apathy is defined as a lack of motivation for goal-directed behavior or thought^10^ without sadness or hopelessness. Although apathy was long considered to be linked with depression, it is now conceptualized as an independent neuropsychiatric symptom.^10,11^ In a study^12^ of 2354 patients with DAT, apathy was identified as an independent symptom cluster in addition to hyperactivity, psychosis, and affective symptoms (including depression). Recently, apathy has been classified as a multidimensional deficit with emotional, behavioral, and cognitive domains.^10^ Although loss of interest might be partly associated with depression, emotional apathy with symptoms of emotional neutrality seems not to be associated with depression.^13,14^

The neural basis of apathy in DAT has been examined in several neuroimaging studies.^15^ Among other regions, the dopamine-related frontostriatal circuitry including the anterior cingulate cortex and the prefrontal cortex seem to be involved.^16,17^ From a neurotransmitter perspective, low levels of dopamine are associated with reduced motivational and reward-driven behavior and have been linked to apathy.^18^ Similarly, an inverse association has been reported between dopamine and noradrenaline transporter binding in the ventral striatum with higher apathy scores in patients with Parkinson disease.^19^

On the basis of these findings, effective treatment of apathy may be achieved with a pharmacological compound that increases dopaminergic and noradrenergic neurotransmission. Support for this approach comes from a recent randomized clinical trial^20^ of 60 male veterans with DAT that showed a beneficial effect of the dopamine and noradrenaline reuptake inhibitor methylphenidate on apathy. In that study, patients with DAT with and without co-occurring symptoms of depression were included. Methylphenidate has a short half-life and can only be prescribed according to controlled substances laws in some countries. Therefore, other drugs might even be more suited for the treatment of apathy in DAT. Bupropion is a dopamine and noradrenaline reuptake inhibitor licensed for use as an antidepressant. Bupropion has been shown to increase psychomotor activity in a mouse model of DAT.^21^ Case reports in frontotemporal dementia^22^ and poststroke apathy^23^ supported bupropion as a potentially effective drug for the treatment of apathy.

Here, we report a 12-week, multicenter, double-blind, placebo-controlled, randomized clinical trial that tested the effect of bupropion on apathy in patients with DAT. Patients with concomitant depressed mood were excluded to avoid potential effects on apathy by improvement of depressed mood.

## Methods

### Study Design

This study was designed as a 12-week, multicenter, double-blind, placebo-controlled, randomized clinical trial in patients with mild-to-moderate DAT with clinically relevant apathy and without clinically relevant symptoms of depressed mood. The trial was conducted in a psychiatric and neurological outpatient setting between July 2010 and July 2014 in Germany. The study was conducted according to the Declaration of Helsinki and was approved by the institutional review board of each participating center. The study was monitored by an independent Data and Safety Monitoring Board. This study follows the Consolidated Standards of Reporting Trials (CONSORT) reporting guideline. The complete study protocol can be found in Supplement 1.

Before screening, all patients and their caregivers gave written informed consent to the full study protocol. In case a patient was incapable of providing informed consent because of progressed cognitive impairment, a legal guardian substituted. After an initial screening period of 4 weeks and the baseline assessment, visits were scheduled at 4, 8, and 12 weeks after baseline. In addition, 2 safety visits were conducted at 2 and 6 weeks after baseline.

### Inclusion and Exclusion Criteria

Patients were recruited only in outpatient settings. The diagnosis of DAT was established according to criteria of the National Institute of Neurological and Communicative Disorders and Stroke and the Alzheimer’s Disease and Related Disorders Association (probable AD).^24^ Patients were eligible for the study if they were aged 55 to 90 years, had a Mini-Mental State Examination (MMSE) score between 10 and 25, and had a caregiver who was willing to participate as a study partner.

The presence of clinically relevant apathy was operationalized by applying the revised Marin and Starkstein apathy in AD criteria.^25^ In addition, all patients had to score at least 4 points on the apathy item of the Neuropsychiatric Inventory (NPI).^26^ A score of 4 points or higher was considered to indicate a clinically meaningful neuropsychiatric symptom on each domain of the NPI. To investigate the effect of bupropion specifically on apathy and to prevent contamination by effects on depressed mood, patients who either fulfilled the major depressive episode *Diagnostic and Statistical Manual of Mental Disorders* (Fourth Edition) criterion of depressed mood or scored 4 points or higher on the dysphoria and depression item of the NPI were excluded.

Patients who either were not receiving antidementia drug treatment or who had been receiving stable treatment with acetylcholinesterase inhibitors and/or memantine for at least 3 months before baseline were included. Patients with dementia other than DAT were excluded. Patients with severe somatic or psychiatric conditions that had led to inpatient hospital treatment during the last 6 months before study participation were not considered. Because of the particular potential adverse effects and contraindications of bupropion, patients with a history of seizures, cerebral tumors, severe traumatic brain injury, or clinically relevant kidney or liver dysfunction were excluded. Patients with unstable diabetes were also excluded. Concomitant treatment with drugs that potentially lower the seizure threshold or that are metabolized by cytochrome P450 isoenzyme 2D6 or that may interfere with bupropion metabolism was prohibited. Also, continuous treatments with antipsychotic or antidepressant medication, benzodiazepines, dopaminergic medication, monoamine oxidase inhibitors, or amantadine within the last 4 weeks before study participation were exclusion criteria.

### Study Treatment Groups

After baseline, patients were randomized to receive either bupropion or placebo. The initial dose of bupropion was 150 mg once daily or 1 identical placebo dose, respectively. If the tolerability was sufficient, the dose was increased to 150 mg twice daily or placebo twice daily after 4 weeks. In case of intolerable adverse effects, the dose could be decreased again to 150 mg once daily or 1 placebo dose and continued at that dose until the end of the study. Study adherence was measured by medication count and caregiver feedback at each follow-up visit. Randomization was conducted at baseline by the Center for Clinical Studies, University of Cologne, and included a random block design with blocks of variable length providing a balanced increase of participating patients in both treatment groups (bupropion:placebo = 1:1). Randomization was stratified for comedication with donepezil or galantamine because both are metabolized by cytochrome P450 isoenzyme 2D6, which is mildly inhibited by bupropion.

### Efficacy Measures

The primary outcome measure was the mean change in the Apathy Evaluation Scale–Clinician Version (AES-C) score.^27,28^ This scale consists of 18 questions that are answered on a Likert scale from 1 to 4, resulting in a range of 18 to 72 points. Higher scores indicate greater apathy. The German version of the scale has been shown to be reliable and valid.^28^ In addition, the AES-C can be divided into an emotional subfactor (range, 2-8), a behavioral subfactor (range, 5-20), a cognitive subfactor (range, 8-32), and a subfactor that includes other items (range, 3-12). The subfactors of the AES-C were analyzed as secondary outcome measures. Additional secondary outcome measures were the NPI total score (range, 0-144),^26^ the Caregiver Distress Scale of the NPI (range, 0-60),^26^ the Alzheimer Disease Cooperative Study–Activities of Daily Living Scale (range, 0-78),^29^ self and proxy ratings of the Quality of Life-AD Scale (QoL-AD) (range, 13-53),^30^ the cognitive subscale of the Alzheimer Disease Assessment Scale (ADAS-Cog) (range, 0-80),^31^ the MMSE (range, 0-30),^32^ and the Montgomery-Asberg Depression Rating Scale (MADRS) (range, 0-60).^33^ Higher values on the NPI, the NPI Caregiver Distress Scale, the MADRS, and the cognitive subscale of the Alzheimer Disease Assessment Scale reflect worse outcomes. Higher values on the Alzheimer Disease Cooperative Study–Activities of Daily Living Scale, MMSE, and the QoL-AD reflect better outcomes.

The NPI and the NPI Caregiver Distress Scale were assessed at the screening visit, at baseline, and at 4, 8, and 12 weeks of follow-up. The MMSE was administered at the screening visit and at 4, 8, and 12 weeks of follow-up. All other scales including the AES-C were assessed at baseline at the 4-week, 8-week, and 12-week visits.

### Safety

Safety was examined by assessing vital signs, electrocardiogram, change in comedication, physical examination, and assessment of suicidality by clinical examination at each visit. Adverse events (AEs) were monitored throughout the study.

### Statistical Analysis

All statistical analyses were performed at the Institute of Medical Statistics and Bioinformatics at the University of Cologne, Germany. Initial power calculation suggested a sample size of 216 patients (108 bupropion and 108 placebo) to be enrolled to test for a 5-point difference on the primary outcome (AES-C) of which an SD of 11.5 points has been reported in a comparable patient sample.^28^ This would equal an effect size of Cohen *d* = 0.43. The target number included an estimated dropout rate of 20%. Because of the low recruitment rate, the study sponsor, the funding agency, the responsible biometrician, the ethics committee, and the Data and Safety Monitoring Board agreed to insert an interim analysis based on 50% of the planned subjects (ie, based on 108 participants plus 2 exclusions at baseline). For the same reason and also because of the missing trend-level effect in favor of bupropion, the parties later agreed to prematurely terminate the study (ie, declaring the interim analysis as the final one). A trend effect was considered a greater but nonsignificant effect of bupropion on apathy compared with placebo. The clinical study protocol was amended accordingly. On the basis of the reduced sample size of 108 participants (ie, 54 per group), an effect size of 0.54 (ie, 6.3 points between-group difference divided by 11.5 points within-group SD) could still be detected with 80% power at 2-sided type I error of 5% (by the 2-sample *t* test).

The 2 treatment groups were compared with χ^2^ tests for categorical variables and with independent *t* tests for continuous variables on demographic characteristics and baseline clinical characteristics, including primary and secondary outcome parameters. The intention-to-treat (ITT) population was used to perform primary and secondary efficacy analyses. The ITT population consisted of all patients who received at least 1 dose of the study medication (bupropion or placebo) and who took part in at least 1 follow-up visit with completion of the AES-C score. The missing completely at random analysis for missing values was applied.^34^ The primary efficacy analysis (mixed-effect model repeated measures) tested the difference of the individual change of the AES-C total scores between baseline and the 12-week visit between the 2 treatment groups. The mixed-effect model repeated-measures model was corrected for the baseline AES-C score, site, and comedication with donepezil or galantamine. Both donepezil and galantamine are partly metabolized by cytochrome P450 isoenzyme 2D6, which is inhibited by bupropion. Thus, bupropion may affect the plasma concentration of donepezil and galantamine, which may, in turn, change the mild effect of these compounds on apathy. Rivastigmine and memantine are not metabolized by cytochrome P450 isoenzyme 2D6. The secondary efficacy analyses were conducted in the same manner and stratified for the baseline score, site, and comedication with donepezil or galantamine. The safety analysis compared the number of AEs and serious AEs between the treatment groups applying the χ^2^ test. The same procedure was conducted with the per-protocol population, which included all patients who completed all study visits and who adhered to study medication. Statistical significance was set at 2-sided *P* < .05. The statistical analysis (comparison of mean values) of secondary outcomes essentially is descriptive (ie, without controlling the familywise error). All statistical analyses were performed using SPSS statistical software version 22 (IBM Corp). Data analyses were performed between August 2018 and August 2019.

## Results

**Figure 1.**
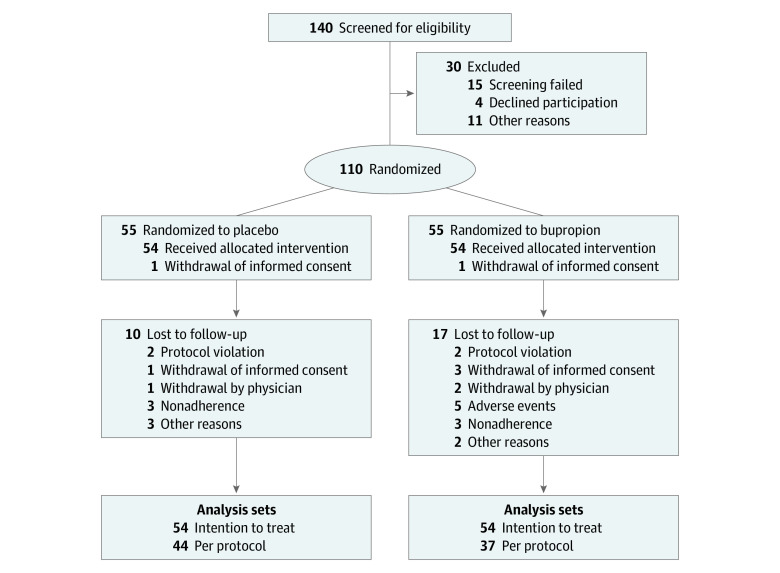
Flow Diagram of Patient Progress Through the Trial of Bupropion vs Placebo for the Treatment of Apathy in Alzheimer Disease

Of 140 screened patients, 108 were included in the ITT analysis (mean [SD] age, 74.8 [5.9] years; 67 men [62%]). The study flow is depicted in Figure 1. Of the 140 screened patients, 110 were randomized. Before the first application of the study medication, 1 patient withdrew in each treatment group. Of the 108 patients who participated, 10 in the placebo group and 17 in the bupropion group dropped out of the study. Thus, the ITT population consisted of 54 patients in each treatment group, whereas 44 patients in the placebo group and 37 in the bupropion group completed the study per protocol. Results for the per-protocol population are reported in eTable 1, eTable 2, eTable 3, and the eFigure in Supplement 2.

Demographic and screening characteristics and baseline outcome measures are listed in Table 1. There were no substantial differences between the groups concerning demographic and screening data (for the bupropion group vs the placebo group, mean [SD] age, 75.3 [5.5] years vs 74.4 [6.3] years; mean [SD] education, 9.7 [3.1] years vs 9.9 [2.6] years; mean [SD] NPI apathy score, 7.2 [2.7] vs 7.4 [2.4]; mean [SD] NPI depression score, 0.6 [1.1] vs 0.4 [0.9]; median [interquartile range], NPI depression score, 0.0 [0.0-1.0] vs 0.0 [0.0-0.0]; MMSE score >18, 34 patients [63%] in each group). Outcome measures at baseline were comparable between the 2 groups (mean [SD] AES-C score, 52.2 [8.7] vs 50.4 [8.2]), except for the proxy rating of the QoL-AD, which was higher (equals better quality of life) in the placebo group than in the bupropion group (mean [SD] score, 32.9 [4.5] vs 30.9 [5.2]).

**Table 1.** Demographic and Screening Characteristics and Outcome Parameters of Patients in Both Treatment Groups (Intention to Treat Population) at Baseline

Characteristic	Mean (SD)			*P* value^a^
	All patients (N = 108)	Bupropion group (n = 54)	Placebo group (n = 54)	
Continuous variables				
Age, y	74.8 (5.9)	75.3 (5.5)	74.4 (6.3)	.39
Education, y	9.8 (2.9)	9.7 (3.1)	9.9 (2.6)	.66
NPI item apathy score	7.3 (2.6)	7.2 (2.7)	7.4 (2.4)	.68
NPI item depression score				
Mean (SD)	0.5 (1.0)	0.6 (1.1)	0.4 (0.9)	.25
Median (interquartile range)	0.0 (0.0-0.0)	0.0 (0.0-1.0)	0.0 (0.0-0.0)	
Categorical variables, patients, No. (%)				
Male	67 (62.0)	31 (57.4)	36 (66.7)	.32
Mini-Mental State Examination score >18	68 (63.0)	34 (63.0)	34 (63.0)	>.99
Consent form signed by legal representative	28 (25.9)	14 (25.9)	14 (25.9)	>.99
Comedication with donepezil or galantamine	72 (66.7)	34 (63.0)	38 (70.4)	.41
Primary outcome parameter, Apathy Evaluation Scale-Clinician version total score	51.3 (8.5)	52.2 (8.7)	50.4 (8.2)	.25
Secondary outcome parameters, score				
Apathy Evaluation Scale-Clinician version				
Cognition	24.0 (4.1)	24.5 (4.0)	23.5 (4.2)	.22
Behavior	13.2 (2.5)	13.4 (2.7)	12.9 (2.3)	.35
Emotion	4.9 (1.5)	5.1 (1.5)	4.8 (1.4)	.26
Other	9.2 (1.9)	9.3 (1.8)	9.2 (2.0)	.68
NPI total	16.2 (9.3)	16.4 (8.5)	16 (10.1)	.83
NPI distress total	8.0 (5.8)	8.0 (6.1)	7.9 (5.5)	.91
Alzheimer Disease Cooperative Study–Activities of Daily Living	52.0 (16.8)	50.1 (17.4)	54 (16.1)	.23
Alzheimer Disease Assessment Scale–Cognitive Subscale	35.3 (12.1)	35.2 (12.6)	35.4 (1.6)	.93
Mini-Mental State Examination	19.3 (4.1)	19.4 (4.1)	19.3 (4.3)	.85
Montgomery-Asberg Depression Rating Scale	9.2 (5.8)	9.9 (5.7)	8.4 (5.7)	.12
Quality of Life in Alzheimer Disease Scale	37.6 (4.3)	37 (4.9)	38.1 (3.5)	.21
Quality of Life in Alzheimer Disease Scale proxy	31.9 (4.9)	30.9 (5.2)	32.9 (4.5)	.03

Abbreviation: NPI, Neuropsychiatric Inventory.

^a^ *P* values for continuous data were calculated with the *t* test for independent groups. *P* values for categorical data were calculated with the χ^2^ test.

**Figure 2.**
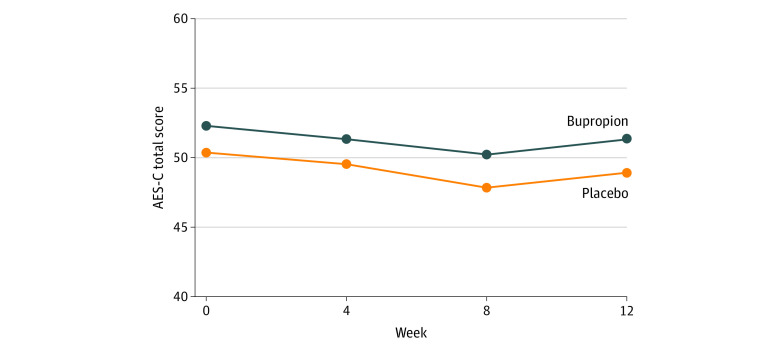
Apathy Evaluation Scale-Clinician (AES-C) Total Score Over Time in Patients With Alzheimer Dementia Receiving Bupropion or Placebo (Intention to Treat Population)

Results for the primary outcome measure are shown in Figure 2 and Table 2. There was no statistically significant effect of bupropion compared with placebo on the mean change of the AES-C total score between baseline and 12 weeks (mean change between groups, 2.22; 95% CI, –0.47 to 4.91; *P* = .11). There was numerically greater improvement of the AES-C total score in the placebo group (mean change within group, 2.07; 95% CI, –0.06 to 4.21) than in the bupropion group (mean change within group, –0.14, 95% CI, –2.34 to 2.05).

**Table 2.** Results of the Mixed-Effect Model Repeated Measure (Intention-to-Treat Population)^a^

Parameter and group	Score, mean change (95% CI)		*P* value
	Within groups	Between groups	
Primary outcome parameter, AES-C total			
Bupropion	–0.14 (–2.34 to 2.05)	2.22 (–0.47 to 4.91)	.11
Placebo	2.07 (–0.06 to 4.21)		
Secondary outcome parameter			
AES-C cognition			
Bupropion	0.21 (–0.89 to 1.31)	1.03 (–0.33 to 2.39)	.14
Placebo	1.24 (0.18 to 2.30)		
AES-C behavior			
Bupropion	–0.12 (–0.89 to 0.64)	0.60 (–0.34 to 1.55)	.21
Placebo	0.48 (–0.27 to 1.23)		
AES-C emotional			
Bupropion	–0.54 (–0.94 to –0.14)	0.54 (0.04 to 1.03)	.03
Placebo	0.00 (–0.39 to 0.38)		
AES-C other			
Bupropion	0.03 (–0.49 to 0.55)	0.28 (–0.37 to 0.93)	.39
Placebo	0.31 (–0.19 to 0.82)		
Neuropsychiatric Inventory total			
Bupropion	0.24 (–2.56 to 3.04)	5.52 (2.00 to 9.04)	.003
Placebo	5.75 (3.02 to 8.49)		
Neuropsychiatric Inventory Caregiver Distress Scale total			
Bupropion	–0.66 (–2.14 to 0.82)	3.03 (1.18 to 4.87)	.002
Placebo	2.36 (0.91 to 3.82)		
Alzheimer Disease Cooperative Study–Activities of Daily Living			
Bupropion	2.81 (0.47 to 5.15)	–2.92 (–5.89 to 0.06)	.05
Placebo	–0.11 (–2.37 to 2.16)		
Alzheimer Disease Assessment Scale–Cognitive Subscale			
Bupropion	–1.53 (–3.93 to 0.87)	–0.27 (–3.26 to 2.73)	.86
Placebo	–1.80 (–4.15 to 0.55)		
Mini-Mental State Examination			
Bupropion	0.05 (–1.08 to 1.17)	–0.45 (–1.84 to 0.94)	.53
Placebo	–0.40 (–1.51 to 0.71)		
Montgomery-Asberg Depression Rating Scale			
Bupropion	–0.79 (–2.04 to 0.45)	2.10 (0.53 to 3.67)	.009
Placebo	1.31 (0.10 to 2.51)		
Quality of Life in Alzheimer Disease Scale			
Bupropion	1.23 (0.10 to 2.36)	–1.66 (–3.01 to –0.31)	.02
Placebo	–0.43 (–1.52 to 0.66)		
Quality of Life in Alzheimer Disease Scale proxy			
Bupropion	0.08 (–1.17 to 1.32)	–2.03 (–3.58 to –0.47)	.01
Placebo	–1.95 (–3.13 to –0.76)		

Abbreviation: AES-C, Apathy Evaluation Scale–Clinician version.

^a^ Table shows difference between baseline and 12 weeks between treatment groups corrected for the baseline score, site, and comedication with donepezil and galantamine.

Results for the secondary outcome parameters are listed in Table 2. The difference in the mean change in the emotional subfactor of the AES-C was statistically significant (mean change between groups, 0.54; 95% CI, 0.04 to 1.03; *P* = .03) with a worsening in the bupropion group. There were statistically significant differences for the mean change between baseline and 12 weeks for the NPI total score (mean change between groups, 5.52; 95% CI, 2.00 to 9.04; *P* = .003) and the NPI Caregiver Distress scale (mean change between groups, 3.03; 95% CI, 1.18 to 4.87; *P* = .002), with a greater reduction in neuropsychiatric symptoms in the placebo group than in the bupropion group (mean change within groups, 5.75 [95% CI, 3.02 to 8.49] vs 0.24 [95% CI, –2.56 to 3.04]) and a higher reduction of caregiver’s distress in the placebo group than in the bupropion group (mean change within groups, 2.36 [95% CI, 0.91 to 3.82] vs –0.66 [95% CI, –2.14 to 0.82]). There was a statistically significant difference in the change on the MADRS between groups (mean change between groups, 2.10; 95% CI, 0.53 to 3.67; *P* = .009) reflecting a mild improvement of subthreshold depressive symptoms in the placebo group (mean change within group, 1.31; 95% CI, 0.10 to 2.51) and a slight worsening the bupropion group (mean change within group, –0.79; 95% CI, –2.04 to 0.45). Finally, the QoL-AD self and proxy rating scales showed an improvement in quality of life in the placebo group (mean changes within group, self rating, –0.43 [95% CI, –1.52 to 0.66]; proxy rating, –1.95 [95% CI, –3.13 to –0.76]) and a worsening in the bupropion group (mean changes within group, self rating, 1.23 [95% CI, 0.10 to 2.36]; proxy rating, 0.08 [95% CI, –1.17 to 1.32]). The mean change between groups was statistically significant for the QoL-AD self rating (mean change between groups, –1.66; 95% CI, –3.01 to –0.31; *P* = .02) and proxy rating (mean change between groups, –2.03; 95% CI, –3.58 to –0.47; *P* = .01). No statistically significant mean changes were found for the AES-C subfactors cognition (mean change between groups, 1.03; 95% CI, –0.33 to 2.39; *P* = .14), behavior (mean change between groups, 0.60; 95% CI, –0.34 to 1.55; *P* = .21), and other (mean change between groups, 0.28; 95% CI, –0.37 to 0.93; *P* = .39), for the Alzheimer Disease Cooperative Study–Activities of Daily Living Scale (mean change between groups, –2.92; 95% CI, –5.89 to 0.06; *P* = .05), the cognitive subscale of the Alzheimer Disease Assessment Scale (mean change between groups, –0.27; 95% CI, –3.26 to 2.73; *P* = .86), and the MMSE (mean change between groups, –0.45; 95% CI, –1.84 to 0.94; *P* = .53).

The AEs and serious AEs are listed in Table 3; 39 bupropion-treated patients (72.2%) and 33 placebo-treated patients (61.1%) experienced at least 1 AE. Seven patients experienced a serious AE leading to hospitalization (5 in the bupropion group [9.3%] and 2 in the placebo group [3.7%]). All serious AEs were most likely unrelated to the study medication. No deaths occurred. Altogether, 157 AEs occurred in 108 patients. The bupropion group had more AEs per patient than the placebo group (mean [SD], 1.8 [1.8] vs 1.1 [1.2] AEs; median [interquartile range], 1 [0-3] vs 1 [0-2] AEs). Of the 150 nonserious AEs, 94 (62.67%) occurred in bupropion-treated patients and 56 (37.33%) in placebo-treated patients. The most frequent AEs were gastrointestinal symptoms, which occurred more often in the placebo group than in the bupropion group (10 patients [17.2%] vs 6 patients [6.1%]). Other frequent AEs were sleeping difficulties (14 patients [18.9%] total), falls (8 patients [5.1%] total), and unrest or confusion (7 patients [4.5%] each). None of the AEs occurred significantly more often in the bupropion group than in the placebo group.

**Table 3.** Comparison of Adverse and Serious Adverse Events in Patients With Alzheimer Dementia Receiving Bupropion or Placebo (Intention-to-Treat Population)

Adverse event	Patients, No. (%)			*P* value^a^
	All patients (N = 108)	Bupropion group (n = 54)	Placebo group (n = 54)	
Patients with adverse event	72 (66.7)	39 (72.2)	33 (61.1)	.22
Patients with serious adverse event	7 (6.5)	5 (9.3)	2 (3.7)	.24
Hospitalization for suspected lymphoma	1 (1.4)	1 (1.9)	0	
Hospitalization for hypoglycemia	1 (1.4)	1 (1.9)	0	
Hospitalization for syncope	1 (1.4)	1 (1.9)	0	
Hospitalization for urinary tract infection	1 (1.4)	1 (1.9)	0	
Hospitalization for atrial fibrillation	1 (1.4)	1 (1.9)	0	
Hospitalization for coprostasis	1 (1.4)	0	1 (1.9)	
Hospitalization for hematuria	1 (1.4)	0	1 (1.9)	
Adverse events per patient, No.				
Mean (SD)	1.5 (1.6)	1.8 (1.8)	1.1 (1.2)	.04
Median (interquartile range)	1 (0-2)	1 (0-3)	1 (0-2)	
All nonserious adverse events^b^	150 (95.5)	94 (95.0)	56 (96.6)	.11
Gastrointestinal symptoms	16 (10.2)	6 (6.1)	10 (17.2)	.03
Sleeping difficulties	14 (8.9)	10 (10.1)	4 (6.9)	.50
Falls	8 (5.1)	7 (7.1)	1 (1.7)	.14
Unrest or anxiety	7 (4.5)	6 (6.1)	1 (1.7)	.20
Confusion	7 (4.5)	6 (6.1)	1 (1.7)	.20
Hallucinations	5 (3.2)	5 (5.1)	0	.08

^a^ *P* values for categorical data were calculated with the χ^2^ test. *P* values for nonparametric data were calculated with the Mann-Whitney *U* test.

^b^ There were 99 adverse events in the bupropion group and 58 adverse events in the placebo group, for a total of 157 adverse events. The percentages in this section are based on these totals. Listed is the number of nonserious adverse events that occurred in ≥5% of patients receiving bupropion.

## Discussion

In this study, which, to our knowledge, is the largest randomized clinical trial on apathy in DAT so far, treatment with bupropion failed to improve apathy as measured with the AES-C in nondepressed patients with DAT over a period of 12 weeks compared with placebo. Moreover, statistically significant uncorrected differences in mean change between the treatment groups were found for the NPI total score, NPI distress score, MADRS, and the QoL-AD favoring the placebo group.

Our findings were comparable to those of a recent randomized clinical trial^35^ of 40 nondepressed patients with Huntington disease, where bupropion was not effective in the treatment of apathy as rated by an informant compared with placebo. In that study,^35^ a general positive effect on apathy was observed by trial participation.

Support for the dopaminergic hypothesis of apathy came from a recent randomized clinical trial^20^ with methylphenidate that showed a significant improvement of apathy. However, that study^20^ did not control for depression, and almost 60% of the participants had concomitant depression. Therefore, the decrease in apathy might also be associated in part with effects of methylphenidate on depression, which has been reported in geriatric depression.^36^ Another difference was the inclusion of patients with moderate dementia in our study (MMSE score, 10-25) compared with the methylphenidate trial (MMSE score, ≥18).^20^ In less affected patients with mild dementia and mild cognitive impairment, response to treatment might be better.^20^

The use of the AES-C as the primary outcome measure of this trial has to be discussed. At the time when the study was conceptualized (2008-2009), the AES-C was the most used and standard measure for apathy in DAT. Recently, it has been shown that the scale is a 1-dimensional test, with a substructure that does not include all dimensions of apathy.^37,38^ Therefore, future studies should use more advanced measures, such as the Dimensional Apathy Scale, that better discriminate between apathy and overlap symptoms of depression, as well as between different apathy subdimensions.^39^ Because of the lack of an established pharmacological treatment of apathy in DAT, nonpharmacological therapies, such as the use of information and communication technologies or occupational therapy, are still first-line recommendations.^40^

### Strengths and Limitations

A strength of this double-blind, placebo-controlled, randomized clinical trial was the exclusion of patients with clinically relevant depression. This allowed the assessment of bupropion on apathy without contamination of the treatment effect by changes in depressed mood.

A limitation of this study was that we failed to reach the estimated required sample size of 216 patients and that we stopped the trial after an interim analysis that found no beneficial effect of bupropion on apathy. This entailed a relevant loss of statistical power. However, because of the lack of an effect in favor of bupropion on apathy, a full recruitment of 216 patients would not have resulted in a positive finding. The identification of suitable patients was difficult throughout the study. Although apathy occurs independently of depressed mood in patients with DAT, most potentially eligible patients in the respective outpatient clinics showed dysphoric mood in addition to apathy. Also, nondepressed patients with apathy often did not seem to be distressed in a way that they wished to participate in a clinical study. As a result of their apathy, volunteerism for trial-related activities was often low, which might have led to a selection bias. In addition, we cannot exclude that lack of motivation was associated with dropout. In some cases, caregivers considered apathy helpful to maintain their daily caregiving activities and thus did not feel the need for treatment of apathy. Furthermore, the study physicians had to be extensively trained to actively seek for signs of apathy during clinical evaluation, because apathy was usually not reported. According to the present analysis, which is both interim and final, however, there was no evidence for any effect of bupropion on apathy.

In the placebo group, we observed improvements in scores on the NPI, the NPI Caregiver Distress Scale, the MADRS, and the Qol-AD that were not present in the bupropion group. One reason for this difference might be the higher rate of AEs in patients receiving bupropion compared with those receiving placebo in the ITT population. This might also explain the higher dropout rate in the bupropion group compared with the placebo group (per-protocol population, 37 of 54 patients in the bupropion group vs 44 of 54 patients in the placebo group). In the per-protocol population, the mean number of AEs was not different between the 2 treatment groups, suggesting that patients who experienced AEs dropped out of the study. We could not exclude the possibility that data were missing not at random (ie, as a result of unknown circumstances related to the trial treatments). However, according to Molenberghs et al,^34^ the mixed-effect model repeated measures analysis performed is stable even if the assumption of missing not at random is violated.

## Conclusions

In this study, bupropion was not superior to placebo for the treatment of apathy in patients with DAT and apathy in the absence of clinically relevant depression. Because of the substantial impact of apathy on patients’ quality of life,^3^ more randomized clinical trials are needed to find an efficient treatment. Future studies are required to further analyze the pathophysiological mechanisms and neurotransmitter alterations underlying apathy in DAT.

## Notes

Supplement 1. **Trial Protocol**

Supplement 2. **eTable 1.** Demographics, Screening Characteristics and Outcome Parameters of Patients With Bupropion Versus Placebo (PP Population) **eFigure.** Apathy Evaluation Scale-Clinician Total Score Over Time in Patients With Alzheimer’s Disease Receiving Bupropion or Placebo (PP Population) **eTable 2.** Results of the Mixed-Effect Model Repeated Measure (PP population): Difference Between Baseline and 12-Weeks Between Treatment Groups Corrected for the Baseline Score, Site and Co-medication With Donepezil/Galantamine **eTable 3.** Comparison of Adverse and Serious Adverse Events in DAT Patients Receiving Bupropion or Placebo (PP Population)

Supplement 3. **Data Sharing Statement**
