# Error in Affiliations

**DOI:** 10.1001/jamanetworkopen.2021.14100

**Published:** 2021-05-26

In the Original Investigation titled “Bupropion for the Treatment of Apathy in Alzheimer Disease: A Randomized Clinical Trial,”^1^ published May 28, 2020, there was an error in the affiliation of co-author Andreas Fellgiebel. The correct affiliation is Center for Mental Health in Old Age, Landeskrankenhaus, Department of Psychiatry and Psychotherapy, University Medical Center, Mainz, Germany. This article has been corrected.^1^
