# Supplementary material for: Bupropion for the Treatment of Apathy in Alzheimer Disease: A Randomized Clinical Trial
Source: JAMA Netw Open. 2020 May 28;3(5):e206027. doi: 10.1001/jamanetworkopen.2020.6027 (PMC7256670; doi:10.1001/jamanetworkopen.2020.6027)
Supplement: Supplement 1. — Trial Protocol [file jamanetwopen-3-e206027-s001.pdf]

STUDY PROTOCOL – SYNOPSIS

A 12-week, multicenter, randomized, double-blind, placebo-controlled trial of Bupropion for the treatment of apathy in Alzheimer's Dementia

Short title: Apa-AD

EudraCT: 2007-005352-17

Sponsor-ID: apaalz01

Phase: III

Investigational drug: **Bupropion**

Date: 06.10.2011

**Sponsor**	**Rheinische Friedrich-Wilhelms-University Bonn**<br>**Regina-Pacis-Weg 3**<br>**53012 Bonn**<br><br>**Represented by:**<br>**Prof. Dr. med. Frank Jessen**<br>**Department of Psychiaty und Psychotherapy**<br>**University of Bonn**<br>**Sigmund-Freud-Str. 25**<br>**53105 Bonn**<br>**Tel.: +49-228-287-11109**<br>**Fax: +49-228-287-16097**<br>**e-mail: Jessen@uni-bonn.de**
Principal Investigator	**Prof. Dr. med. Frank Jessen** (See above)
Study title	A 12-week, multicenter, randomized, double-blind, placebo-controlled trial of Bupropion for the treatment of apathy in Alzheimer's Dementia
**Investigational drug**	Bupropion (Elontril®)
Indication	Apathy in Alzheimer's dementia
Time frame	Submission of the study to the institutional review board and state authorities: June 2009<br>Study accepted by institutional review board and state authorities: January 2010<br>Inclusion of first patient: July 2010

	Inclusion of last patient: November 2012<br>Completion of last patient: February 2013<br>Close out: March 2013<br>Completion of statistical analysis: May 2013<br>Final report: June 2013
Phase:	III
Setting / sites:	Multi-center, national study (~~11~~ 13 sites in Germany)
Primary Outcome	Change score on the German version of the apathy evaluation scale (AES)
Secondary Outcomes	Change on the German version of the neuropsychiatric inventory (NPI)<br>Change on the German version of the NPI caregiver distress scale<br>Change on the German version of the Alzheimer's Disease Consortium Activities of daily living scale (ADCS-ADL)<br>Change on the German version of the Quality of Live AD-Scale (QoL-AD)<br>Change on the German version of the Resources Utility Questionnaire (RUD)<br>Change on the German version of the Alzheimer's Disease Assessment Scale (ADAScog)<br>Change on the German version of the Mini-Mental State Examination (MMSE)
Design	Two-armed, randomized, double-blind, placebo-controlled trial

Number of patients	216 patients (108 patients in the bupropion group, 108 in the placebo group)<br>Recruitment period: 18 months<br>Interim analysis after the inclusion of 108 patients added
Inclusion Criteria	• Informed consent for study participation<br>• mild to moderate Alzheimer's dementia (NINCDS/ADRDA-criteria)<br>• age between 55 and 90 years (including)<br>• Mini-Mental State Examination (MMSE): 10 to ~~23~~ **25** points (including)<br>• ≥4 points on the apathy items of the neuropsychiatric inventory (NPI)<br>• Fulfillment of the revised Starkstein Apathy criteria<br>• outpatient setting<br>• Availability of a caregiver<br>• no concomitant treatment with anti-dementia drugs or stable treatment with anti-dementia drugs for the last three months without changes in the medication plan prior to inclusion<br>• for females: Postmenopausal<br>• cerebral brain scan (cCT, cMRI), which is consistent with the diagnosis of probable Alzheimer's disease
Exclusion Criteria	• Dementias with other etiology than Alzheimer's disease (e.g. vascular dementia, Lewy Body dementia, frontotemporal dementia, mixed forms)

	• major depressive disorder, defined by the modified DSM IV criteria<br>(depressed mood must be present)<br>• ≥4 points on the NPI depression item<br>• present alcohol- or benzodiazepine addiction<br>• continuous treatment (present or during the last four weeks) with neuroleptic or antidepressive medication (including Hypericum perforatum)<br>• continuous treatment (present or during the last four weeks) with dopaminergig drugs or Amantadine<br>• continuous treatment (present or during the last four weeks) with benzodiazepines<br>• continuous treatment (present or during the last four weeks) with mono-amino-oxidase inhibitors<br>• known sensitivity towards bupropion<br>• severe psychiatric disease during the last 6 months that resulted in hospitalization<br>• history of suicide attempts<br>• acute psychotic symptoms<br>• severe physical illness which does not allow the participation throughout the 12 week treatment period<br>• history of seizures<br>• history of central nervous system tumors<br>• history of severe brain injury with cerebral lesions<br>• clinically relevant kidney failure and/or hepatic failure

	• concomitant treatment with medication that lower the seizure threshold (e.g. neuroleptics, antidepressive medication, Malaria medication, tramadol, theophylline, systemic steroids in higher dosages, Chinolone, sedative antihistamines)<br>• concomitant treatment with medication which is metabolized by<br>Cytochrom P450-Isoenzyme 2D6 (e.g. the following beta-blocker Metoprolol, Propanolol, Timolol, Carvediol, Nebivolol, Type 1C antiarrhythmics such as Propafenone, Flecainid), excluding Donepezil and<br>Galantamine<br>• concomitant treatment with medication which might interfere with bupropion metabolization (e.g. Carbamazepine, Phenytoine, Valproat, Ritonavir, Lopinavir)<br>• poorly controlled but medically treated Diabetes mellitus<br>• concomitant treatment with stimulants or appetite suppressants<br>• Participation in another clinical study during the last three months<br>• history of lactulose intolerance<br>• present suicidality
**Investigational drug, dose, route of administration**	Study drug: Bupropion<br>The verum group receives 1×150 mg Bupropion orally. After four weeks, the first efficacy assessment will be performed.

	In case of good tolerability, Bupropion can be increased to 300mg (2 tablets) at this visit, which will be the final dose throughout the trial. If the patient does not tolerate 300mg, he/she may be down titrated to 150mg (1 tablet), which will then be the fixed dose throughout the study.
Duration of treatment:	12 weeks
Placebo**, dose, route of administration**	Placebo<br>The placebo group receives 1 tablet of placebo orally. After four weeks, the placebo group receives 2 tablets.<br>If the patient does not tolerate 2 tablets, he/she may be down titrated to 1 tablet, which will then be the fixed dose throughout the study.
Efficacy criteria	Efficacy:<br>• mean difference between the bupropion group and the placebo group on the change on the Apathy Evaluation Scale after 12 weeks<br>Safety:<br>• documentation of side effects and adverse events
Statistician	Prof. Dr. Martin Hellmich<br>Institute of Medical Statistics and Computational Biology, Faculty of Medicine, University of Cologne, Cologne, Germany<br>Kerpener Straße 62<br>50937 Cologne, Germany<br>Tel. 0221-478 6503

	Fax 0221 478 6520<br><br>E-mail: Martin.Hellmich@uni-koeln.de
Statistical methods	Analysis of covariance (ANCOVA) was used to analyze the differences between the two treatment groups regarding the primary outcome (individual change on the AES between baseline and 12 weeks). The ANCOVA was stratified for the AES-baseline score, site, and co-medication with donepezil/galantamine.<br><br>Level of significance was set at 5% (two-tailed).<br><br>The intention-to-treat (ITT) population was used for primary and secondary efficacy analyses.<br>The per-protocol (PP) population was used for sensitivity analyses.
GCP-conformity:	This trial will be carried out in accordance with the internationally accepted "Good Clinical Practice-Guidelines (ICH-GCP)" as well as in accordance with the national law (AMG, GCP-V)
Funding	German Ministry of Education and Research (Bundesministerium für Bildung und Forschung, BMBF); grant number: 01KG0909)
