# Supplementary material for: Bupropion for the Treatment of Apathy in Alzheimer Disease: A Randomized Clinical Trial
Source: JAMA Netw Open. 2020 May 28;3(5):e206027. doi: 10.1001/jamanetworkopen.2020.6027 (PMC7256670; doi:10.1001/jamanetworkopen.2020.6027)
Supplement: Supplement 2. — eTable 1. Demographics, Screening Characteristics and Outcome Parameters of Patients With Bupropion Versus Placebo (PP Population) eFigure. Apathy Evaluation Scale-Clinician Total Score Over Time in Patients With Alzheimer’s Disease Receiving Bupropion or Placebo (PP Population) eTable 2. Results of the Mixed-Effect Model Repeated Measure (PP population): Difference Between Baseline and 12-Weeks Between Treatment Groups Corrected for the Baseline Score, Site and Co-medication With Donepezil/Galantamine eTable 3. Comparison of Adverse and Serious Adverse Events in DAT Patients Receiving Bupropion or Placebo (PP Population) [file jamanetwopen-3-e206027-s002.pdf]

Supplementary Online Content

Maier F, Spottke A, Bach J-P, et al. Bupropion for the treatment of apathy in Alzheimer disease: a randomized clinical trial. *JAMA Netw Open*. 2020;3(5):e206027. doi:10.1001/jamanetworkopen.2020.6027

eTable 1. Demographics, Screening Characteristics and Outcome Parameters of Patients With Bupropion Versus Placebo (PP Population)

eFigure. Apathy Evaluation Scale-Clinician Total Score Over Time in Patients With Alzheimer's Disease Receiving Bupropion or Placebo (PP Population)

eTable 2. Results of the Mixed-Effect Model Repeated Measure (PP population): Difference Between Baseline and 12-Weeks Between Treatment Groups Corrected for the Baseline Score, Site and Co-medication With Donepezil/Galantamine

eTable 3. Comparison of Adverse and Serious Adverse Events in DAT Patients Receiving Bupropion or Placebo (PP Population)

This supplementary material has been provided by the authors to give readers additional information about their work.

© 2020 Maier F et al. *JAMA Network Open.*

SUPPLEMENT DATA – Per-protocol Analysis

eTable 1. Demographics, Screening Characteristics and Outcome Parameters of Patients With Bupropion Versus Placebo (PP Population)

	**All patients (N=81)**		**Bupropion (N=37)**		**Placebo (N=44)**		
**Continuous variables**	**mean**	**SD**	**mean**	**SD**	**mean**	**SD**	**p[‡]**
Age (yrs)	74.1	5.7	74.3	5.5	73.9	6	.75
Education (yrs)	9.6	2.6	9.1	2.3	10.1	2.8	.09
NPI Item Apathy	7.4	2.8	7.3	3.1	7.4	2.6	.95
NPI Item Depression	0.5	0.9	0.6	1.1	0.3	0.8	.24
**Categorical variables**	**N**	**%**	**N**	**%**	**N**	**%**	
Males	51	63	22	59.5	29	65.9	.55
MMSE > 18	49	60.5	23	62.2	26	59.1	.78
Consent form signed by legal representative	22	27.2	10	27	12	27.3	.98
Co-Medication with donepezil /galantamine	56	69.1	24	64.9	32	72.7	.45
**Primary Outcome Parameter**	**mean**	**SD**	**mean**	**SD**	**mean**	**SD**	**p**
AES-Clinician total	51.3	8.5	52.4	9	50.3	8.1	.26
**Secondary Outcome Parameter**							
AES-Clinician Cognition	24.0	4.1	24.6	4.0	23.5	4.1	.23
AES-Clinician Behavior	13.1	2.5	13.5	2.8	12.8	2.2	.21
AES-Clinician Emotion	5	1.5	5.2	1.6	4.8	1.4	.21
AES-Clinician Other	9.2	1.8	9.1	1.8	9.2	1.9	.87
NPI total	15.6	9.3	15.7	8.5	15.6	10.1	.98
NPI Distress total	7.7	4.9	7.9	4.8	7.5	5.1	.71
ADCS-ADL	52.4	16.4	50.9	16.6	53.7	16.3	.44
ADAS-Cog	35.1	12.2	34.7	12.6	35.5	11.9	.78
MMSE	19.2	4.2	19.3	4.2	19.1	4.3	.83
MADRS	9.2	6	10.6	5.7	8	6	.05
QoL-AD	37.4	3.9	36.2	4.3	38.4	3.2	**.01**
QoL-AD proxy	32.2	5.0	30.6	5.0	33.5	4.7	**.01**

AES, Apathy evaluation scale; NPI, neuropsychiatric inventory, ADCS-ADL, Alzheimer's Disease Cooperative Study – Activities of daily living; ADAS-Cog, Alzheimer's disease assessment scale – cognitive subscale; MMSE, Mini-Mental State Examination; MADRS, Montgomery Asberg Depression Rating Scale; QoL-AD, Quality of life in Alzheimer's Disease Scale.

‡ P values for continuous data: t-test for independent groups, P values for categorical data: Chi^2-test

© 2020 Maier F et al. *JAMA Network Open.*

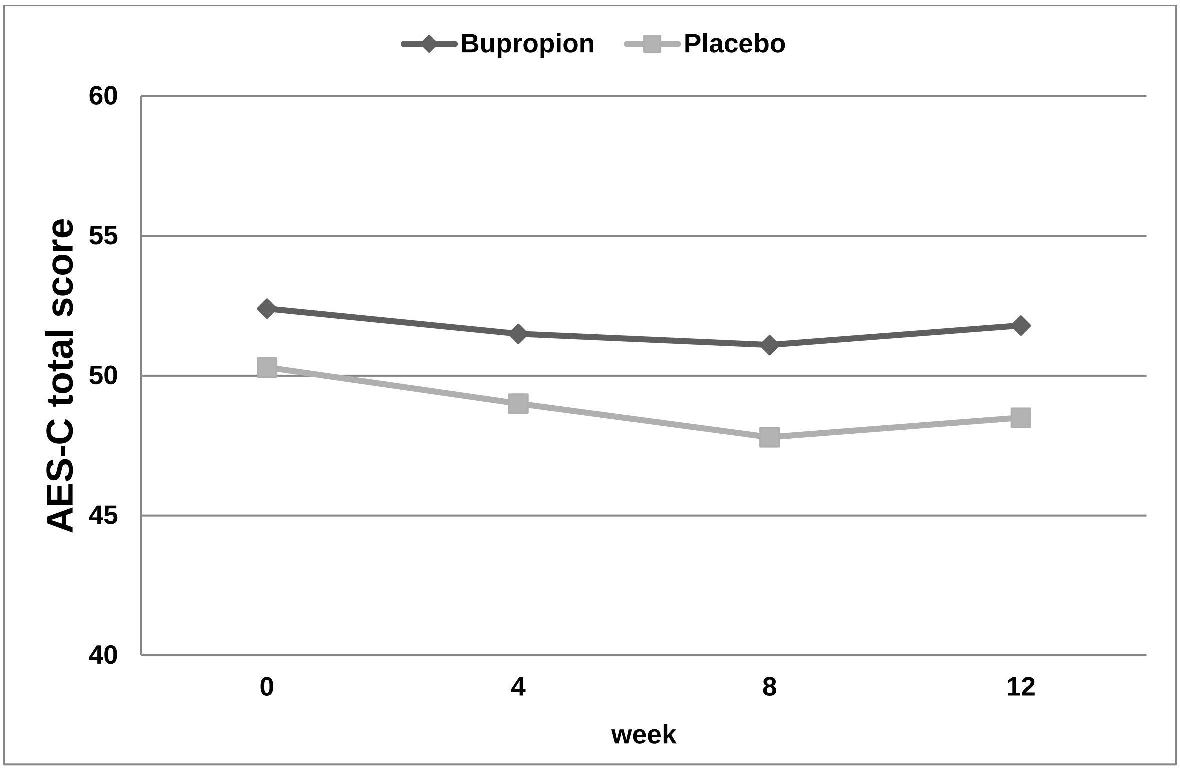

eFigure. Apathy Evaluation Scale-Clinician Total Score Over Time in Patients With Alzheimer's Disease Receiving Bupropion or Placebo (PP Population)

© 2020 Maier F et al. *JAMA Network Open.*

eTable 2. Results of the Mixed-Effect Model Repeated Measure (PP population): Difference Between Baseline and 12-Weeks Between Treatment Groups Corrected for the Baseline Score, Site and Co-medication With Donepezil/Galantamine

		**in Groups**			**between Groups**			
		**Mean**	**95%-CI**		**Mean**	**95%-CI**		**P**
**Primary outcome parameter**	**Group**	**Change**	**lower**	**upper**	**Change**	**lower**	**upper**	**value**
AES-C total score	Bupropion	0.52	-1.96	3.01				
	Placebo	2.42	0.02	4.82	1.90	-1.20	4.99	0.225
**Secondary outcome parameter**								
AES-C Cognition	Bupropion	0.58	-0.65	1.81				
	Placebo	1.27	0.09	2.45	0.69	-0.85	2.23	0.376
AES-C Behavior	Bupropion	0.14	-0.77	1.05				
	Placebo	0.63	-0.25	1.51	0.49	-0.65	1.63	0.390
AES-C Emotional	Bupropion	-0.52	-0.93	-0.10				
	Placebo	0.14	-0.27	0.54	0.65	0.12	1.18	**0.016**
AES-C Other	Bupropion	0.02	-0.55	0.59				
	Placebo	0.31	-0.24	0.87	0.29	-0.43	1.02	0.423
NPI total	Bupropion	-0.10	-3.09	2.89				
	Placebo	6.28	3.39	9.17	6.38	2.56	10.19	**0.001**
NPI Distress total	Bupropion	-0.69	-2.31	0.92				
	Placebo	2.35	0.78	3.92	3.04	0.98	5.10	**0.004**
ADCS-ADL	Bupropion	2.60	-0.08	5.28				
	Placebo	-0.37	-2.93	2.20	-2.96	-6.41	0.48	0.091
ADAS-Cog	Bupropion	-1.01	-3.91	1.88				
	Placebo	-2.14	-4.89	0.61	-1.13	-4.82	2.57	0.545
MMSE	Bupropion	-0.06	-1.35	1.23				
	Placebo	-0.28	-1.54	0.97	-0.23	-1.86	1.41	0.784
MADRS	Bupropion	-1.13	-2.55	0.29				
	Placebo	1.16	-0.20	2.53	2.29	0.47	4.12	**0.014**
QoL-AD	Bupropion	0.77	-0.41	1.95				
	Placebo	-1.07	-2.16	0.04	-1.84	-3.32	-0.35	**0.016**
QoL-AD proxy	Bupropion	0.37	-1.01	1.76				
	Placebo	-2.22	-3.51	-0.93	-2.59	-4.34	-0.84	**0.004**

© 2020 Maier F et al. *JAMA Network Open.*

AES, Apathy evaluation scale; NPI, neuropsychiatric inventory, ADCS-ADL, Alzheimer's Disease Cooperative Study – Activities of daily living; ADAS-Cog, Alzheimer's disease assessment scale – cognitive subscale; MMSE, Mini-Mental State Examination; MADRS, Montgomery Asberg Depression Rating Scale; QoL-AD, Quality of life in Alzheimer's Disease Scale.

© 2020 Maier F et al. *JAMA Network Open.*

eTable 3. Comparison of Adverse and Serious Adverse Events in DAT Patients Receiving Bupropion or Placebo (PP Population)

	All patients (N=81)		Bupropion (N=37)		Placebo (N=44)		
	**N**	**%**	**N**	**%**	**N**	**%**	**p‡**
**Patients with adverse event**	53	65.4	26	70.3	27	61.4	.40
**Patients with serious adverse event**	5	6.2	3	8.1	2	4.6	.88
Hospitalization for suspected lymphoma	1	1.2	1	2.7	0	0	
Hospitalization for hypoglycemia	1	1.2	1	2.7	0	0	
Hospitalization for syncope	1	1.2	1	2.7	0	0	
Hospitalization for coprostasis	1	1.2	0	0	1	2.3	
Hospitalization for hematuria	1	1.2	0	0	1	2.3	
**All adverse events**	**N=106**		**N=60**		**N=46**		
	**Mean**	**SD**	**Mean**	**SD**	**Mean**	**SD**	**p‡**
Number of adverse events	1.3	1.5	1.5	1.7	1.0	1.2	.16
	**N**	**%**	**N**	**%**	**N**	**%**	
All unserious adverse events	101	95.3	57	95.0	44	95.7	.43
Gastrointestinal symptoms	15	14.9	6	10.5	9	20.5	.16
Sleeping difficulties	8	7.9	5	10.1	3	6.8	.73
Falls	5	5	4	7.1	1	2.3	.28
Unrest/Anxiety	4	4	3	6.1	1	2.3	.45
Confusion	4	4	4	6.1	0	0	.07
Hallucinations	3	3	3	5.1	0	0	.12
Infection	9	8.9	3	5.1	6	13.6	.14

Listed is the number of unserious adverse events that occurred in ≥ 5% of patients receiving bupropion.

‡ P values for categorical data: Chi^2-test; P values for nonparametric data: Mann Whitney U-Test

© 2020 Maier F et al. *JAMA Network Open.*
