# Supplementary material for: Bupropion for the Treatment of Apathy in Alzheimer Disease: A Randomized Clinical Trial
Source: JAMA Netw Open. 2020 May 28;3(5):e206027. doi: 10.1001/jamanetworkopen.2020.6027 (PMC7256670; doi:10.1001/jamanetworkopen.2020.6027)
Supplement: Supplement 3. — Data Sharing Statement [file jamanetwopen-3-e206027-s003.pdf]

Data Sharing Statement

Maier. Bupropion for the Treatment of Apathy in Alzheimer Disease.
JAMA Netw Open. Published May 29, 2020.
10.1001/jamanetworkopen.2020.6027

Data
Data available: Yes
Data types: Deidentified participant data
How to access data: Email to frank.jessen@uk-koeln.de
When available: With publication

Supporting Documents
Document types: None

Additional Information
Who can access the data: Researchers whose proposed use of the data has been approved
Types of analyses: For other research such as meta-analyses
Mechanisms of data availability: With a signed data access agreement
